# DIFFERENTIAL EFFECTS OF ADULT CHILDREN’S HEALTH PROBLEMS ON PRIMARY AND SECONDARY CAREGIVING FOR PARENTS

**DOI:** 10.1093/geroni/igad104.1624

**Published:** 2023-12-21

**Authors:** Catherine Stepniak, J Jill Suitor, Megan Gilligan

**Affiliations:** Purdue University, West Lafayette, Indiana, United States; Purdue University, West Lafayette, Indiana, United States; University of Missouri, Columbia, Missouri, United States

## Abstract

Adult children often serve as primary and secondary caregivers to their aging parents as they face greater needs for assistance. However, it could be expected that the flow of caregiving support from adult children to parents might be disrupted if adult children are facing their own serious health challenges. Surprisingly, the literature provides no clear picture of the way in which children’s health moderates their provision of care to older parents. In the present study, we shed new light on this question using quantitative and qualitative data collected from 156 mothers ages 75-85 regarding assistance provided by their 586 adult children as part of the Within-Family Differences Study. Additionally, we will explore if there are differences based on timing of the children’s health problems, the types of health problems (physical health vs. psychological health), and gender of the adult children. Quantitative analyses showed that adult children with serious health problems were just as likely as their healthy siblings to be a primary or secondary caregiver to their mothers. Qualitative data revealed the complex emotional and practical cost-benefit analyses mothers and their adult children with health problems engaged in to maintain reciprocal support exchanges. Our findings underscore the importance of using mixed-method approaches to understand the ways in which the provision of care to parents is shaped by adult children’s own health status.

